# Health Outcomes in Patients Using No-Prescription Online Pharmacies to Purchase Prescription Drugs

**DOI:** 10.2196/jmir.2236

**Published:** 2012-12-06

**Authors:** Theodore J Cicero, Matthew Stephen Ellis

**Affiliations:** ^1^School of MedicineDepartment of PsychiatryWashington University in St. LouisSaint Louis, MOUnited States

**Keywords:** prescription drugs, health care quality, access, evaluation, health policy, substance-related disorders

## Abstract

**Background:**

Many prescription drugs are freely available for purchase on the Internet without a legitimate prescription from a physician.

**Objective:**

This study focused on the motivations for using no-prescription online pharmacies (NPOPs) to purchase prescription drugs rather than using the traditional doctor-patient-pharmacy model. We also studied whether users of NPOP-purchased drugs had poorer health outcomes than those who obtain the same drug through legitimate health care channels.

**Methods:**

We selected tramadol as a representative drug to address our objective because it is widely prescribed as an unscheduled opioid analgesic and can easily be purchased from NPOPs. Using search engine marketing (SEM), we placed advertisements on search result pages stemming from the keyword “tramadol” and related terms and phrases. Participants, who either used the traditional doctor-patient-pharmacy model to obtain tramadol (traditional users, n=349) or purchased it on the Web without a prescription from their local doctor (ie, nontraditional users, n=96), were then asked to complete an online survey.

**Results:**

Respondents in both groups were primarily white, female, and in their mid-forties (nontraditional users) to upper forties (traditional users). Nearly all nontraditional users indicated that their tramadol use was motivated by a need to treat pain (95%, 91/96) that they perceived was not managed appropriately through legitimate health care channels. A majority of nontraditional users (55%, 41/75) indicated they used NPOPs because they did not have access to sufficient doses of tramadol to relieve pain. In addition, 29% (22/75) of nontraditional users indicated that the NPOPs were a far cheaper alternative than seeing a physician, paying for an office visit, and filling a prescription at a local pharmacy, which is often at noninsured rates for those who lack medical insurance (37%, 35/96, of NPOP users). The remainder of participants (16%, 12/96) cited other motivations (eg, anonymity) for using NPOPs. In terms of health outcomes, nontraditional users experienced a significantly (*P*<.01) greater number and severity of adverse events, including life-threatening seizures: 7% (7/96) of nontraditional users reported seizures, while none of the traditional users reported seizures.

**Conclusions:**

Although online pharmacies can offer distinct advantages in terms of convenience and cost, users of these “rogue” pharmacies that offer drugs with no prescription or doctor supervision do so at great risk to their health, as evidenced by much higher rates of adverse events. The most logical explanation for these findings is that the lack of physician oversight of dosage schedules, contraindicated conditions, and concomitant medications, were responsible for the increased intensity and frequency of adverse events in the nontraditional users. Although we only examined tramadol, it is logical to postulate that similar results would be observed with dozens of equally accessible prescription drugs. As such, the geometric growth in the use of online pharmacies around the world should prompt intense medical and regulatory discussion about their role in the provision of medical care.

## Introduction

The Internet has evolved into a source of consumer products that were historically only available in “brick-and-mortar” establishments. Recently, there has been growth in the use of the Internet in medical practice, most prominently in the use of online pharmacies to fill physicians’ prescriptions and mail the drug directly to the patient. These pharmacies serve as an important resource for patients, particularly for those who have limited mobility or accessibility needs [1-5]. Unfortunately, this positive use of modern technology has had an unanticipated outcome: the advent of online pharmacies that provide drugs—such as opioid analgesics, antidepressants, cholesterol-management drugs, and erectile dysfunction medications—without a legitimate prescription from a physician [6-11]. In fact, it has been estimated that in the United States alone, 1 in 6 consumers, or roughly 36 million people, have bought or currently buy prescription medications online without a valid prescription [12].

Initially, as the number of no-prescription online pharmacies (NPOPs) [13] increased exponentially in the late 1990s, there was widespread concern that the Internet would serve as a major source of diversion for prescription drug abusers, since many sites offered controlled substances (such as hydrocodone and oxycodone) for purchase [6,14-15]. After Ryan Haight died from an overdose of hydrocodone, allegedly bought over the Internet without a valid prescription, a law bearing his name was passed in 2008 that made it illegal in the United States for NPOPs to sell controlled substances [16]. No matter the reason, research has shown that the Internet has not evolved into a significant source of prescription drugs for the purpose of drug abuse [17-18].

While controlled substances may not be readily accessible from domestic NPOPs [19], other studies have described the availability of many other types of medication through these outlets. These medications include HIV drugs, benzodiazepines, and cholesterol medications, as well as lifestyle drugs, such as diet pills and erectile dysfunction medications. Unlike controlled substances with the potential for abuse, there are no current laws regulating the sale of these other potentially dangerous prescription medications through NPOPs. The main arguments against taking the easiest step, which would be to simply eliminate all online pharmacies, is that they are difficult to close down [4] and that with appropriate controls they can provide consumers advantages in both cost and accessibility [1-5,20]. In fact, one study demonstrated the efficacy of utilizing an accredited online pharmacy to prescribe Viagra. Patients who used the online pharmacy showed similar numbers of side effects and similar treatment efficacy compared to those receiving Viagra through a local pharmacy. They also provided a more complete medical history [21]. To manage the increased use of online pharmacies, however, more regulations are being proposed, including the Online Pharmacy Safety Act and the development of state-run online pharmacy programs [22,23].

In a major effort to steer consumers toward legitimate online pharmacies that are safe to use and away from rogue online pharmacies that pose a potential threat to consumer safety, the National Association of Boards of Pharmacy developed the Verified Internet Pharmacy Practice Sites (VIPPS) program to accredit online pharmacies based on a number of qualifying criteria [24]. Unfortunately, this accreditation is limited to domestic online pharmacies and, as it only approves online pharmacies that require a valid prescription [24], it does not address the larger issue of people seeking medications through NPOPs outside of a typical doctor-patient-pharmacy relationship.

It should be stressed that no matter what legislative controls are adopted, there is a simple way to bypass these restrictions: move the NPOPs offshore, which is rapidly occurring with little government control [7,25-31]. For example, a report by the World Health Organization (WHO) noted that most countries, particularly India and China, which are major loci for NPOPs, have little or no regulation of online pharmacies [11]. This lack of oversight generates a number of safety concerns for NPOP consumers in distribution, information, and medication-related issues. Distribution issues include damaged packaging that exposes pills to light and moisture, shipments that do not meet manufacturer specifications (such as temperature-controlled or insulated packaging), and the ability of the consumer to reorder as many pills as desired [8-10,32-33]. The lack of proper labeling or safety information is common with NPOP-purchased medications and provides consumers with little to no information on dosage scheduling, dosage administration, or potential side effects [9,32-36]. Finally, the medications themselves could be expired, counterfeit, or cut with other substances. Even genuine drugs purchased from NPOPs could lead to a number of adverse events, including death, if the user is unaware of dangerous drug combinations or contraindicated medical conditions [3,5,32,37].

While the recent focus, appropriately, has been placed on the regulation of online pharmacies, there is very little systematic research outside of case reports on two potentially more important basic issues: (1) why consumers use online pharmacies in the place of legitimate medical channels; and (2) with such a variety of safety concerns, why consumers of drugs purchased from NPOPs have worse health outcomes than those who obtain the same the drugs through legitimate healthcare channels. The study described in this paper was designed to address these issues.

## Methods

### Selection of Target Drug

Since most online pharmacies offer dozens of drugs for purchase, we needed to narrow the focus to users of a single representative target drug. Tramadol was selected as the representative drug for this study because it is extensively prescribed (the third most frequently used analgesic [38]) and it is one of the most commonly offered authentic drugs from NPOPs with few restrictions on refills or quantity of tablets offered [8,10]. Tramadol has a demonstrated abuse profile [39-41], but its rate of abuse is not as high as other opioids (hence its noncontrolled status in the Controlled Substances Act). Like all drugs, there is also the potential for adverse side effects that can pose serious health risks. For example, tramadol not only has the potential to produce many of the same adverse events as other opioids (eg, constipation and dependence [39]), but also carries a serious risk of potentially fatal grand mal seizures, which are exacerbated by contraindicated medications and medical conditions [42-44]. In a prior report, we documented the ease of obtaining tramadol over the Internet, the authenticity of which was certified by a chemical analysis [8]. The purchase required the completion of a brief questionnaire that served as a medical examination. Subsequently, a virtual prescription was generated and filled by a pharmacy in Canada. The tramadol was received within 24 hours, and numerous phone calls and emails were received almost immediately to refill the prescription (some offers included up to 400 pills in a single order) and have continued on a monthly basis for over three years thus far.

### Recruitment

It has been widely documented that recruiting and administering surveys over the Internet is an acceptable and beneficial research methodology [45-46]. While these methods provide quick access to thousands of people, they are not easily used to attract a targeted audience. To circumvent this problem, we developed a recruitment program that directly targeted a population of tramadol users with access to the Internet. We utilized search engine marketing (SEM), which is defined as a “form of Internet marketing that seeks to promote websites by increasing their visibility in search engine result pages through the use of paid placement, contextual advertising and paid inclusion [47].” Using Google AdWords and Yahoo Advertising Solutions for this study, we placed short advertisements (eg, “Do You Use Tramadol?”) in the margins of Google and Yahoo search result pages stemming from keyword searches of the term “tramadol” and related terms and phrases (eg, pain relief, Ultram, and buy tramadol online). Thus, our advertisement only appeared to Internet users who had an interest in tramadol or tramadol-related topics, making our target population more likely to include potential participants (ie, users of tramadol). When users clicked the ad, they were automatically directed to an online consent form and the subsequent survey hosted on an institutional website. Subjects were screened to be 18 years of age, users of tramadol in the past 30 days for any reason, and United States residents. Upon completion of the survey, participants were mailed a $50 Visa Check Card for their time.

### Survey Instrument

Since this study represents a preliminary approach into this area of research, no standardized instruments could address all points of inquiry. As such, we developed a descriptive tool centered on our representative drug, in which questions about dosage schedules, adverse events, etc, were specifically related to tramadol. While we developed this descriptive tool to meet the objectives of this pilot study, we hope that the results can provide a basis for a more standardized instrument that can be used to investigate the same objectives for any number of drugs purchased from NPOPs in future studies. Other than demographics, the survey covered a broad variety of topics related specifically to tramadol, including the following: dosage schedule, intended use, comorbidity, legitimate and illegitimate drug use, and adverse events. Participants who listed NPOPs as a source of tramadol were presented with a subset of questions to determine the underlying factors behind their use of online pharmacies.

### Data Analysis

A total of 445 tramadol users qualified for and completed this study. Of these participants, 349 indicated that they received tramadol solely through a valid prescription from their local doctor and filled it at a local pharmacy. These participants are referred to as “traditional users.” The other 96 participants are referred to as “nontraditional users.” This group obtained tramadol from an online pharmacy without a valid prescription from their doctor, and included those who were provided a prescription online by a “virtual” physician. We analyzed data using IBM SPSS Statistics Version 20. We used chi-square and logistical regression analyses to test for significant differences between traditional and nontraditional users at a *P*<.01 level.

## Results

### Demographics

As shown in Table 1, both traditional and nontraditional users were primarily white and female. Traditional users were significantly older than nontraditional users. Looking at various types of health care coverage, 37% (n=35/95) of nontraditional users had no form of insurance, compared to just 16% (n=56/345) of traditional users.

**Table 1:** Demographics and health information for traditional and nontraditional users.

			Traditional users		Nontraditional users		*P* values
			(n=349)		(n=96)		
**Gender, %**							
	Female		67.0		57		.08
**Ethnicity, %**							
	Caucasian		80.2		91		.02
	African American		8.6		3		.07
	Hispanic		4.6		4		.86
	Other		6.6		2		.09
**Age in years, mean (SE)**			47.2 (0.7)		38.5 (1.2)		<.001
**Health care coverage, %**							
	Private		36.8		35		.71
	Dependent		10.4		4		.06
	Medicare/Medicaid		25.2		13		.009
	Military		5.5		1		.07
	Other		5.8		11		.11
	None		16.2		37		<.001

### Use of NPOPs

**Figure 1:**
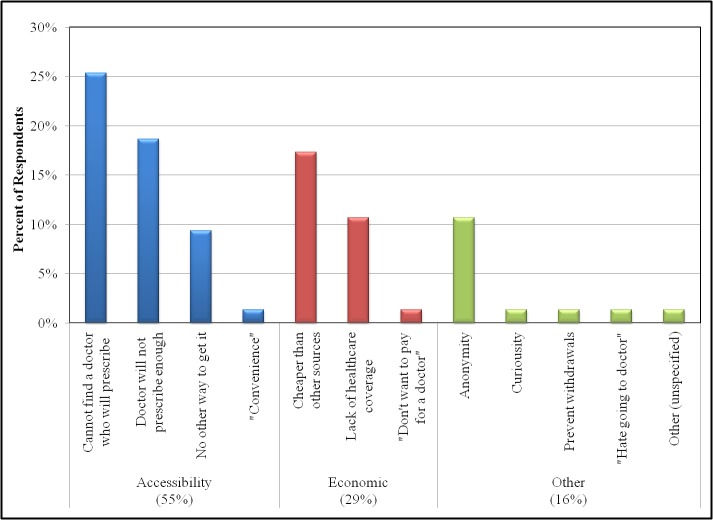
Motivations for using online pharmacies as a source of tramadol reported by nontraditional users (N=96). The values given are the percent of respondents who endorsed a motivation listed or specified a motivation that was not listed (in quotation marks).

We asked nontraditional users what their primary reason was for using online pharmacies in place of other sources (Figure 1): 55% (n=41/75) indicated that they did so for reasons related to accessibility of tramadol (eg, their doctor would not prescribe enough, they could not find a doctor who would prescribe it, or there was no other way to get it), 29% (n=22/75) did so for economic purposes (eg, they had no insurance, their medical plan would not cover it, or it was cheaper than other sources), and 16% (n=12/75) did so for other reasons (eg, anonymity or to prevent withdrawals).

### Tramadol Use

Table 2 shows that nontraditional users were more likely to take the higher dose (100 mg) traditional users. Nontraditional users were also considerably more likely to use tramadol more frequently (5 or more times per week). All traditional users used tramadol for its indicated purpose (ie, to treat pain), with only 2.3% (n=8/349) additionally using tramadol for its euphorigenic properties. Despite using a source outside of legitimate medical channels, the vast majority of nontraditional users (95%, n=91/96) also cited pain as a reason for using tramadol. Of these, 63% (n=60/96) used tramadol for pain only and 32% (n=31/96) used it for both pain and to get high. Just 5% (n=5/96) of nontraditional users indicated that getting high was the main reason for taking tramadol.

**Table 2:** Tramadol use among traditional and nontraditional users.

		Traditional users	Nontraditional users	*P* values
		(n=349)	(n=96)	
**Tramadol dosage, %**				
	25 mg	4.9	5	.91
	50 mg	83.7	73	.02
	100 mg	6.2	19	<.001
	150 mg	5.2	3	.39
**Tramadol frequency, %**				
	1-2 times/week	38.2	26	.03
	3-4 times/week	49.5	29	<.001
	5 or more times/week	12.2	45	<.001
			
**Reasons for using tramadol, %**				
	Only to treat pain	97.7	63	<.001
	Both to treat pain and to get high	2.3	32	<.001
	Only to get high	0.0	5	<.001

### Adverse Events

**Figure 2:**
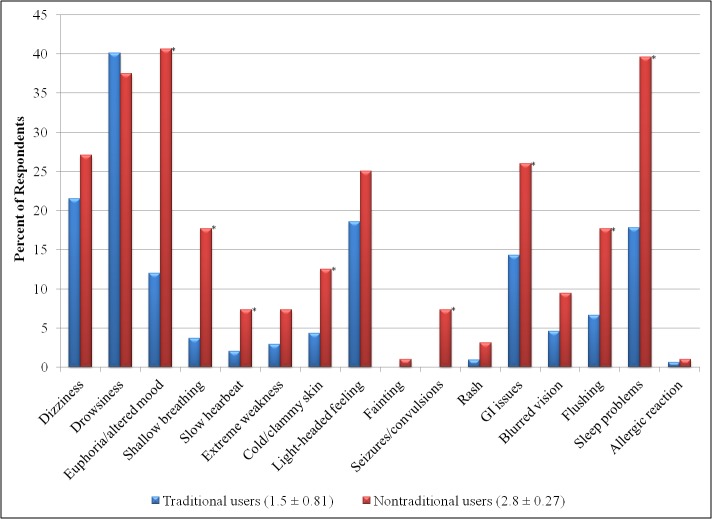
Percent of traditional and nontraditional users who experienced each adverse event while taking tramadol. Asterisks denote a significant difference (*P*<.01) between groups. The legend indicates the mean number of adverse events ± standard error) experienced by both groups.

Figure 2 shows that we found that the difference in the mean number of adverse events experienced by traditional and nontraditional users was significant (*P*<.001). Nontraditional users experienced a much more severe adverse event profile than traditional users (Figure 2). Euphoria, shallow breathing, slow heartbeat, cold/clammy skin, gastrointestinal issues, flushing, and sleep problems all occurred significantly more frequently in the nontraditional users that in the traditional users. Importantly, seizures, which can have potentially fatal outcomes, had an incidence rate of 7% (n=7/96) among nontraditional users, but were not experienced by a single traditional user.

### Physical Dependence

**Figure 3:**
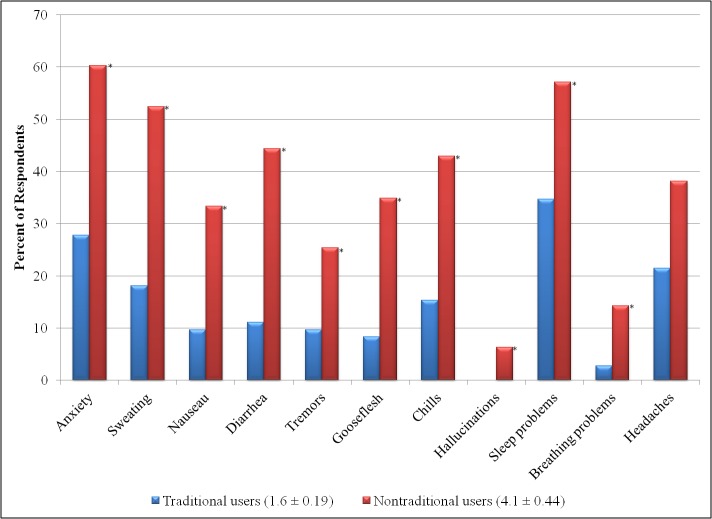
Percent of traditional and nontraditional users who experienced each withdrawal symptom as a result of the abrupt cessation of tramadol. Asterisks denote a significant difference (*P*<.01) between groups. In addition , the legend indicates the mean number of withdrawal symptoms ± standard error experienced by both groups.

Both groups had high rates of suddenly stopping their use of tramadol, but nontraditional users were significantly more likely to cease use abruptly (traditional users: 41.9%, n=144/344; nontraditional users: 66%, n=61/92; *P*<.01). Upon cessation, nontraditional users experienced more severe withdrawal symptoms than traditional users (see Figure 3). We found that the difference in the mean number of withdrawal symptoms experienced by traditional and nontraditional users was significant (*P*<.001).

## Discussion

Our data indicate that those who eschew the typical doctor-patient relationship to obtain tramadol through NPOPs do so primarily for reasons related to cost and accessibility and, most importantly, expose themselves to great health risks. We found that nontraditional users who used NPOPs had much higher rates of all recorded adverse events, particularly life-threatening seizures, than traditional users who obtained a prescription for tramadol from their physician. Seizures are quite rare in normal pain patients being treated with tramadol, observed at a rate of less than 1% [48], and they may be related to the dual effect of tramadol on opioid and adrenergic systems in the brain [49-51]. While the precise mechanisms are unknown, seizures are more prevalent in people who take high doses of tramadol [42,52], have predisposing medical conditions (eg, history of head injuries) [53], or take contraindicated medications (eg, tricyclic antidepressants) [54-55]. Physicians are trained to recognize such predisposing factors, but nontraditional users are likely to be unaware of these potential complications, leading to poor health outcomes. Moreover, we found that nontraditional users experienced much more intense opioid withdrawal symptoms when they stopped taking tramadol. The most logical explanation for these findings is that the *lack of physician oversight* in monitoring dosage schedules, contraindicated conditions, and concomitant medications was responsible for the increased intensity and frequency of adverse events in nontraditional users.

Nearly all nontraditional users in our study indicated that their tramadol use was motivated, at least in part, by a need to treat a health condition (eg, pain) that was not otherwise managed through legitimate health care channels. It was this perception of their unmet medical need (ie, inadequate pain management) that drove them to use NPOPs. This finding raises an important question: Why were normal medical channels shunned in favor of an online pharmacy? There appear to be three distinct motivations for using online pharmacies: (1) inability to pay the costs associated with obtaining a legitimate prescription; (2) limited access to a doctor who would prescribe tramadol or prescribe it at doses sufficient to fully relieve pain; and (3) unwillingness, not inability, to use legitimate medical channels.

With regard to economic motivations, 37% (n=35/95 of NPOP users lacked medical insurance coverage and NPOPs are a less expensive alternative to seeing a physician, paying for an office visit, and filling a prescription at a local pharmacy at noninsured rates. Furthermore, many respondents indicated “no other way to get tramadol” as their main reason for using an NPOP, which suggests there were barriers to accessing a physician, such as cost or the patient’s distance from a medical facility. By far the most commonly reported motivation for using an NPOP was an issue of accessibility: the absence of a physician who was willing to prescribe tramadol either at all or at levels sufficient to meet a patient’s perceived need. There are several possible interpretations of the latter motivation. First, the patient had an unrealistic expectation for “total” pain relief and the physician believed that other drugs would be a suitable alternative to tramadol in providing tolerable pain relief. Second, the physician denied the patient additional tramadol because the doctor incorrectly believed the pain was managed to the extent possible (ie, inadequate pain management). Third, the physician was reluctant to prescribe opioid analgesics, even a weak one such as tramadol, at sufficient levels to adequately relieve pain due to the inherent fear of iatrogenic dependence. At this time, it is unclear which of these was the strongest motivation to use NPOPs, but lack of access to appropriate medical treatment appears to be a major factor. This should not be surprising given the well-documented regional, social, and economic differences in access to medical care in the United States [56-59]. Because of this, there is a large market for the many drugs easily available from online pharmacies, which can best be explained within the context of cost and/or access to appropriate medical care.

While most of the foregoing discussion focused on pain management, 32% (n=31/96) of our population indicated they used an NPOP to buy tramadol for both its euphorigenic and analgesic properties. However, only 5% (n=5/96) reported the Internet as their primary source for tramadol as a drug of abuse. This agrees with a number of studies that show the Internet is not often used as a source of opiates among habitual drug abusers (<5% claim to have obtained their drugs from the Internet [18]). Nevertheless, it needs to be recognized that off-label use to “get high” may serve as one of the motivating factors for the use of NPOPs. In fact, the euphorigenic use of tramadol may explain why a number of respondents indicated “anonymity” as their primary motivator for using NPOPs. It is also possible that some NPOP users, while initially using tramadol for therapeutic purposes, had predisposing factors that led to the development of tramadol misuse or abuse. This euphorigenic use, a health outcome itself, would have led to higher dosages and increased frequency of use, playing a role in the higher rates of adverse events. In a physician-patient relationship, however, a doctor may have recognized predisposing factors for misuse and not prescribed an opioid analgesic or, if already prescribed, recognized the signs of abuse and misuse and switched from tramadol to a less addictive drug.

Although we used tramadol as a prototype in these studies, there is no reason to believe that different results would be observed with dozens of equally accessible prescription drugs obtained through NPOPs that are used without the oversight of a physician. The dangers of overdose and other adverse events with these medications, especially when little to no information about contraindicated medications and medical conditions is included with purchase, have the potential to be more clinically significant with other medications than those we observed with tramadol. As such, the geometric growth in the use of online pharmacies around the world, both legitimate and illegitimate, should prompt intense medical and regulatory discussion about their role, if any, in the provision of medical care.

Currently there are several bills and regulations being discussed to control the use of online pharmacies, some of which ban the use of those located outside of the United States [22,23], but the following two factors need to be considered. First, the passage of online pharmacy regulations that promote verification programs [24], licensure and location disclosures [3], standardized criteria for Internet-based prescriptions [60-61], and a more thorough analysis of the advantages and disadvantages of online health care services (eg, the ability of online pharmacies to detect interactions between medications instantly [5]) may help integrate online pharmacies into health care utilization models. The reality, however, is that regulating these legitimate online pharmacies is likely to have no effect on those using NPOPs. These users have already turned their back on typical medical channels and seem to be able to quickly adapt to any change in access to online pharmacies (eg, shift of NPOPs to foreign countries), and no amount of regulatory oversight would likely change their drug-purchasing behaviors.

Second, so long as a licensed doctor provides a prescription and the pharmacy verifies the legitimacy of the prescription, it would be inappropriate, perhaps unethical, to ban a patient from shopping around to find the most economical and convenient means of filling their prescriptions. Whether this doctor-patient relationship needs to be on a physical basis merits further discussion. Research has shown that email and virtual consultations are just as good, if not better, at capturing patient information necessary for health care decisions [21,62]. However, the old phrase “buyer beware” must be kept in mind, particularly for online pharmacies outside of the United States. Because of aggressive marketing and pricing strategies, as well as the recent shift in patients becoming more involved in their own health care decisions, people using online pharmacies are in danger of unconsciously transforming from patients to consumers, and then back to patients again when they suffer from adverse effects from the use of the drug [13, 63-64]. Patients should be aware of the real possibility that while offshore pharmacies may be cheaper and easier to use, the medications received may not be what was advertised. For this reason, recent US Food and Drug Administration (FDA) and WHO reports have advocated global drug safety, including international cooperation regarding the regulation of online pharmacies [11,31]. Such an effort is badly needed because if one country attempts to ban online pharmacies, most users will simply try a website from another country. Clearly, in addition to regulatory activity, educational efforts are needed to ensure that patients and physicians understand the positive and negative aspects of online pharmacies. Perhaps most importantly, more research is needed to better understand the motivations of people who, despite the availability of legitimate online pharmacies, continue to seek medications using NPOPs.

### Limitations

Inherent in this study are all of the limitations typical of epidemiological and survey research, most notably generalizability and veracity of information gathered. With regard to the latter, most studies indicate that the results obtained from self-administered surveys are comparable to those elicited by trained interviewers. In our study, there were no right or wrong answers. There was no incentive or need to lie about any information because respondents were paid for their participation regardless of their answers. In terms of a biased sample, it is true that our subjects might have greater economic status and certainly more computer literacy than the average person, but these users would most likely to be exposed to advertisements touting online pharmacies.

### Conclusion

Our data suggest that online pharmacies may have a role in supplying prescribed medications because they are convenient and may charge less than traditional brick-and-mortar pharmacies. However, from a public health perspective, the potential benefits of online medical care need to be balanced against the use of unregulated pharmacies that could sell counterfeit or adulterated drugs and the dangers inherent in self-medication without any physician supervision.

## Abbreviations

FDA: US Food and Drug Administration
NPOP: no-prescription online pharmacy
SEM: search engine marketing
VIPPS: Verified Internet Pharmacy Practice Sites
WHO: World Health Organization
